# Supplementary material for: The sperm epigenome does not display recurrent epimutations in patients with severely impaired spermatogenesis
Source: Clin Epigenetics. 2020 May 6;12:61. doi: 10.1186/s13148-020-00854-0 (PMC7204326; doi:10.1186/s13148-020-00854-0)
Supplement: Supplementary file 2 — Additional file 2: Figure S1. A) Age comparison between the normal and the oligozoospermic groups. Wilcoxon rank sum test p-value = 0.8537. B) Age comparison between the 5 NC, 5 AMO and 6 NMO used for the WGBS. Kruskal-Wallis, p-value = 0.0802. Figure S2. Principle component analysis (PCA) of MEST and H19 methylation values obtained by DBS for the 133 sperm samples (Additional file 1: Table S2). Samples with PC1 score below the 95th percentile were considered normally methylated and with PC1 score above the 95th percentile, abnormally methylated. While normal controls (NC) are a homogeneous group of normally methylated samples, oligozoospermic sperm samples were subdivided in two groups according to this PC1 threshold (AMO, abnormally methylated oligozoospermic; NMO, normally methylated oligozoospermic). Point labels are shown for AMO samples only. Figure S3. Methylation levels of the 50 imprinting control regions. Line diagrams showing comparisons between blood (BL1, BL2) and sperm (SP1, SP2) datasets from Laurentino et al. [20] (upper panel), between NC and AMO sperm (middle) and NC and AMO sperm (lower panel) (Additional file 1: Table S4). * Not imprinted according to this data, ** Possible polymorphism. Figure S4. PCA generated for ~8.7 million CpG loci where all samples show methylation values. Only loci with minimum coverage of five in all samples and minimum mapping quality of 10 are considered. Datasets from Laurentino et al. [20] are shown in white (BL1 and BL2 – blood, SP1 and SP2 – sperm), NC sperm samples in teal, AMO sperm samples in orange and NMO sperm in purple. Figure S5. Methylation levels of 2,761 sperm-soma DMRs. Line diagrams showing comparisons between blood (BL1, BL2) and sperm (SP1, SP2) datasets from Laurentino et al. [20] (upper panel), between NC and AMO sperm (middle) and NC and NMO sperm (lower panel). The 2,640 DMRs less methylated in sperm than in blood are towards the left and the 121 DMRs more methylated in sperm than in blood are on [file 13148_2020_854_MOESM2_ESM.pdf]

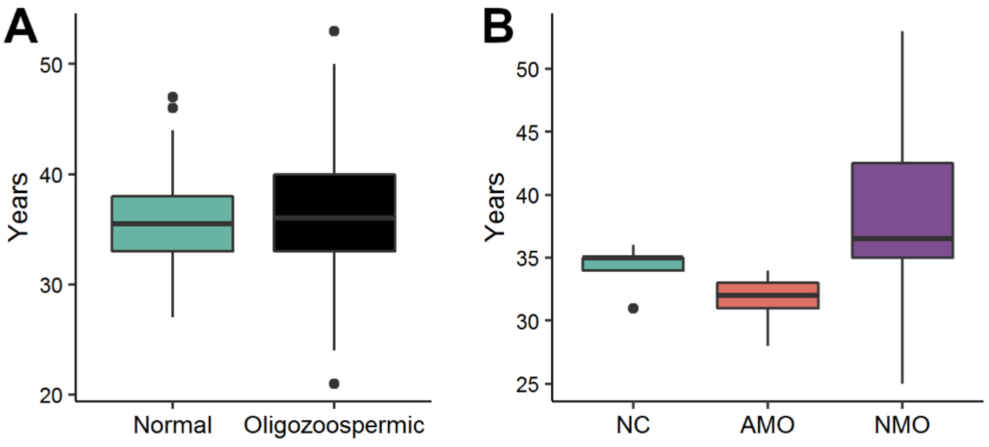

Fig. S1: A) Age comparison between the normal and the oligozoospermic groups. Wilcoxon rank sum test p-value = 0.8537. B) Age comparison between the 5 NC, 5 AMO and 6 NMO used for the WGBS. Kruskal-Wallis, p-value = 0.0802.

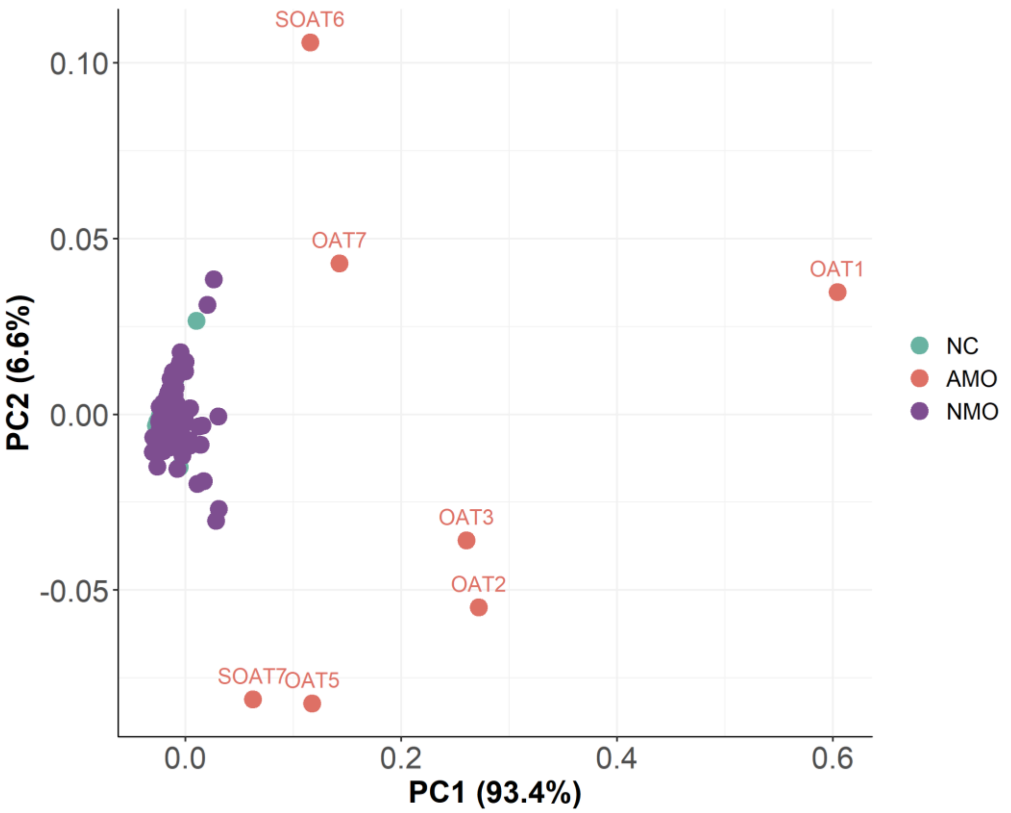

Fig. S2: Principle component analysis (PCA) of *MEST* and *H19* methylation values obtained by DBS for the 133 sperm samples (Additional file 1: Table S2). Samples with PC1 score below the 95th percentile were considered normally methylated and with PC1 score above the 95th percentile, abnormally methylated (Additional file 1: Table S1). While normal controls (NC) are a homogeneous group of normally methylated samples, oligozoospermic sperm samples were subdivided in two groups according to this PC1 threshold (AMO, abnormally methylated oligozoospermic; NMO, normally methylated oligozoospermic). Point labels are shown for AMO samples only.

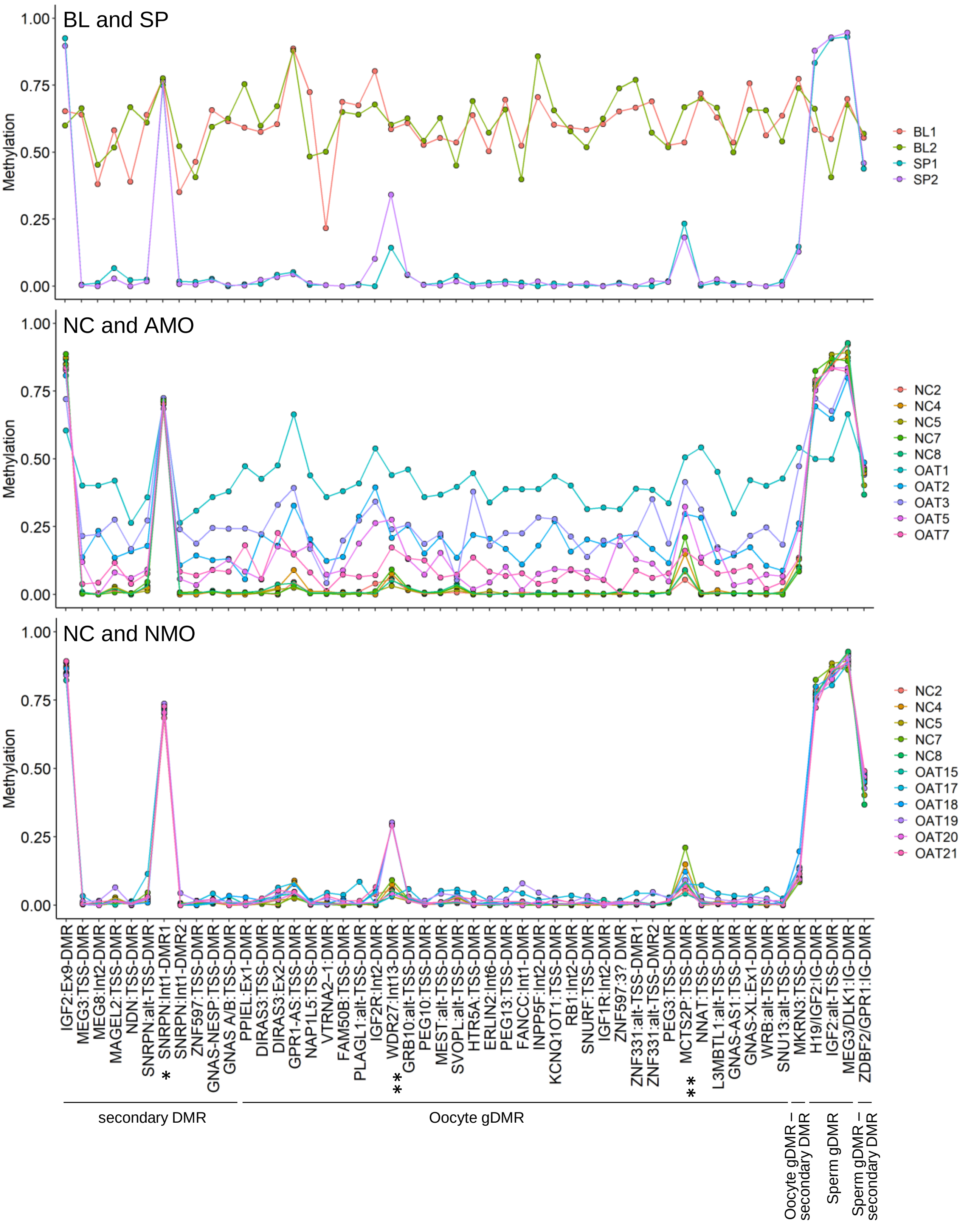

Fig. S3: Methylation levels of the 50 imprinting control regions. Line diagrams showing comparisons between blood (BL1, BL2) and sperm (SP1, SP2) datasets from Laurentino et al. [20] (upper panel), between NC and AMO sperm (middle) and NC and NMO sperm (lower panel) (Additional file 1: Table S4). * Not imprinted according to this data, ** Possible polymorphism.

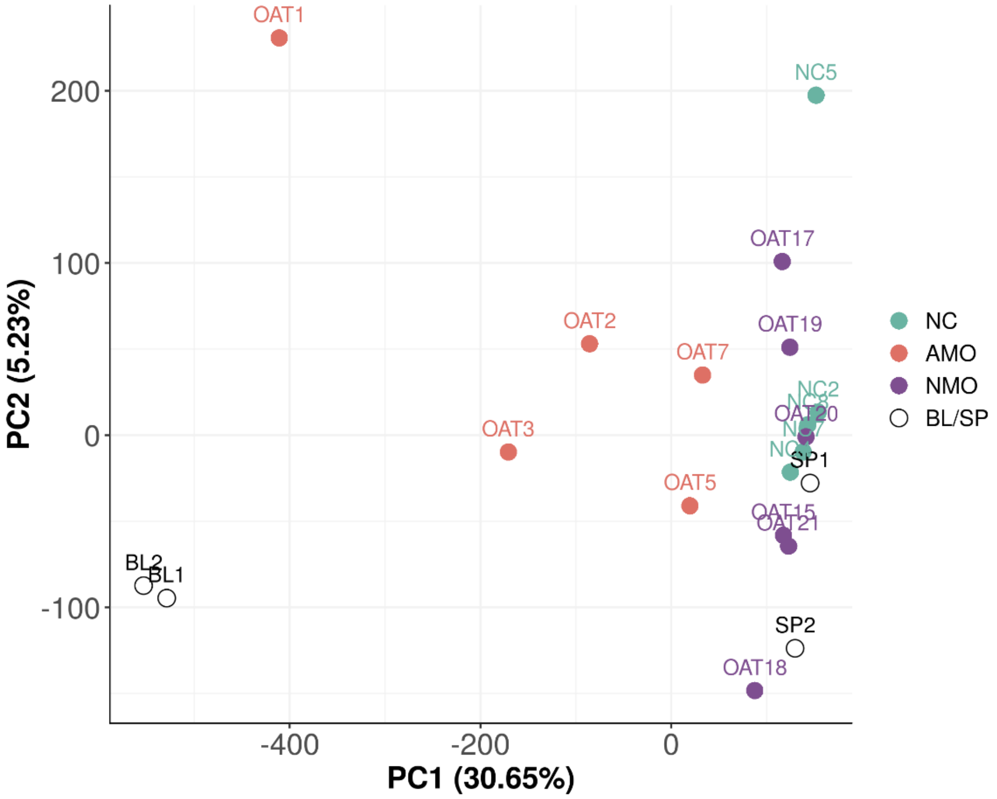

Fig. S4: PCA generated for ~8.7 million CpG loci where all samples show methylation values. Only loci with minimum coverage of five in all samples and minimum mapping quality of 10 are considered. Datasets from Laurentino et al. [20] are shown in white (BL1 and BL2 – blood, SP1 and SP2 – sperm), NC sperm samples in teal, AMO sperm samples in orange and NMO sperm in purple.

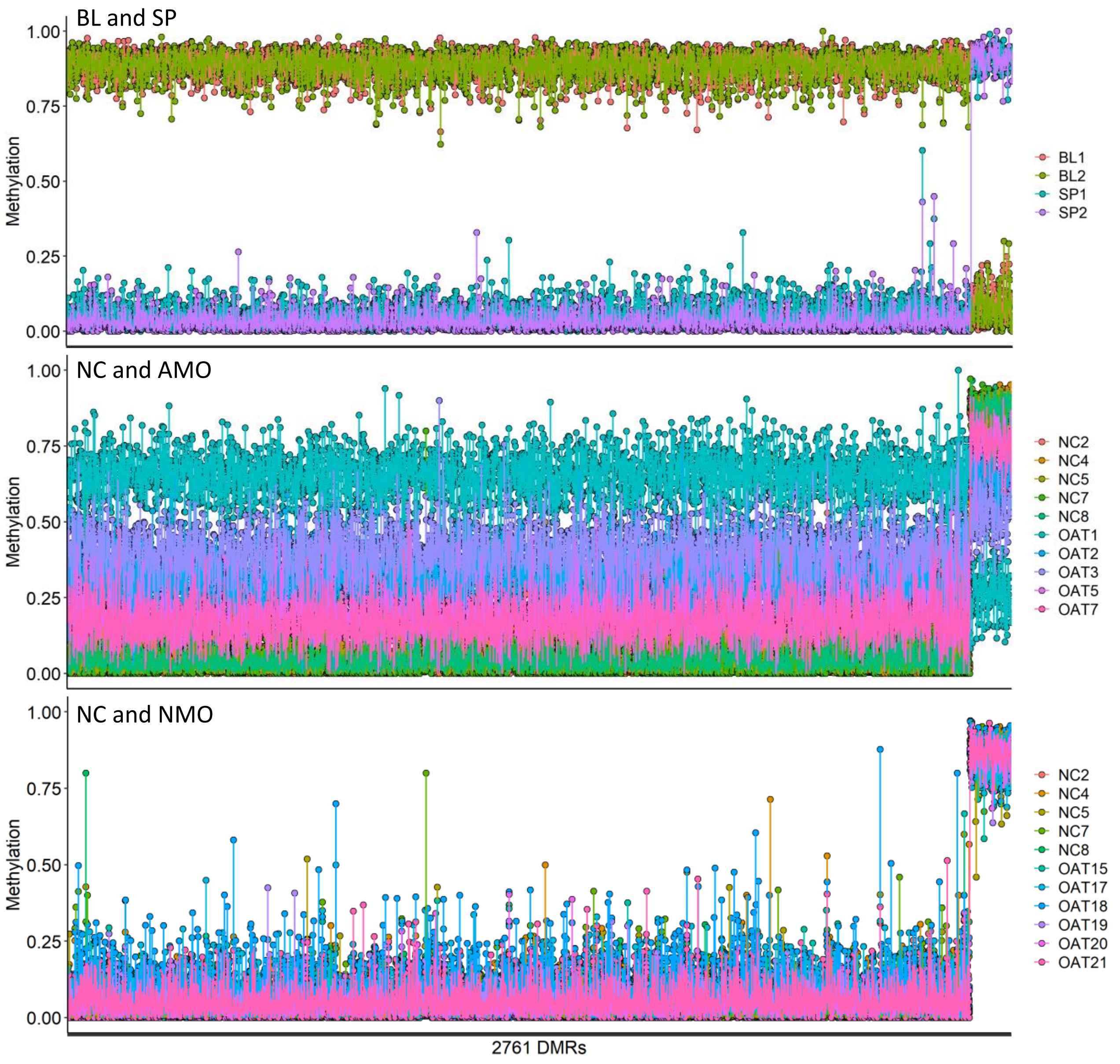

Fig. S5: Methylation levels of 2,761 sperm-soma DMRs. Line diagrams showing comparisons between blood (BL1, BL2) and sperm (SP1, SP2) datasets from Laurentino et al. [20] (upper panel), between NC and AMO sperm (middle) and NC and NMO sperm (lower panel). The 2,640 DMRs less methylated in sperm than in blood are towards the left and the 121 DMRs more methylated in sperm than in blood are on the right (Additional file 1: Table S5).

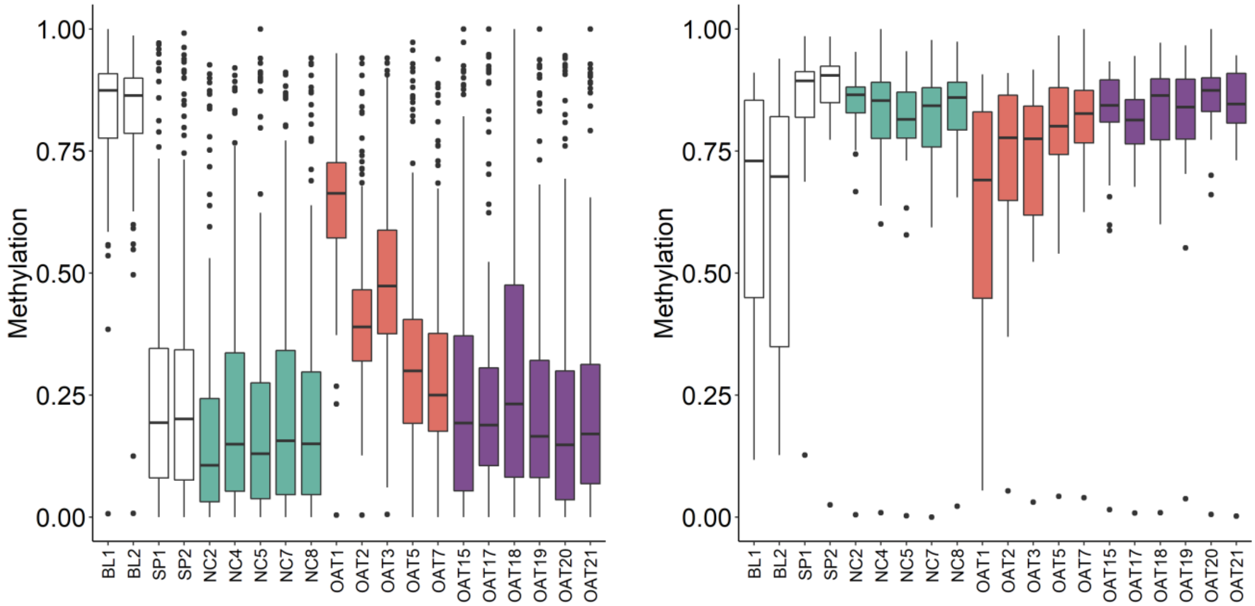

Fig. S6: Methylation levels of the 217 DMRs claimed by Luján et al. [23] to be useful for infertility assessment. Box plots showing the distribution of methylation values for the DMRs stated to be hyper- (190 DMRs, left) or hypomethylated in sperm from oligozoospermic vs. fertile men (20 DMRs, right) (Additional file 1: Table S6). Datasets from Laurentino et al. [20] are shown in white (BL1 and BL2 – blood, SP1 and SP2 – sperm), NC sperm samples in teal, AMO sperm samples in orange and NMO sperm in purple. Box plots elements are defined as follows: center line: median; box limits: upper and lower quartiles; whiskers: 1.5× interquartile range; points: outliers.

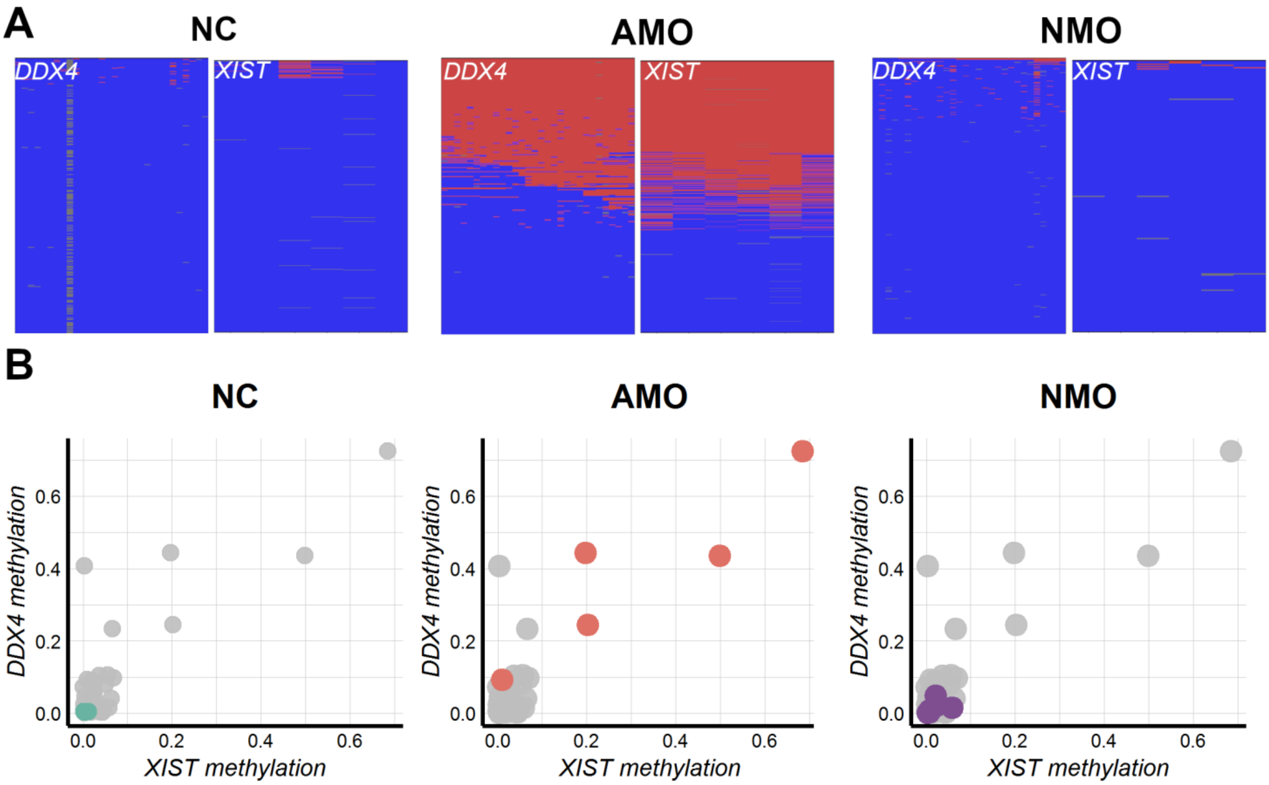

Fig. S7: Validation of *DDX4* and *XIST* methylation levels with deep bisulfite sequencing. A) Example of deep bisulfite sequencing results of *DDX4* and *XIST* in the three groups: NC, AMO and NMO. Each horizontal line of a plot represents a unique sequence read, while each vertical position represents a CpG site (methylated sites in red, unmethylated sites in blue). B) Mean methylation values for *DDX4* and *XIST* in the NC (teal, n=5), AMO (red, n=5) and NMO (purple, n=6) selected for the WGBS.

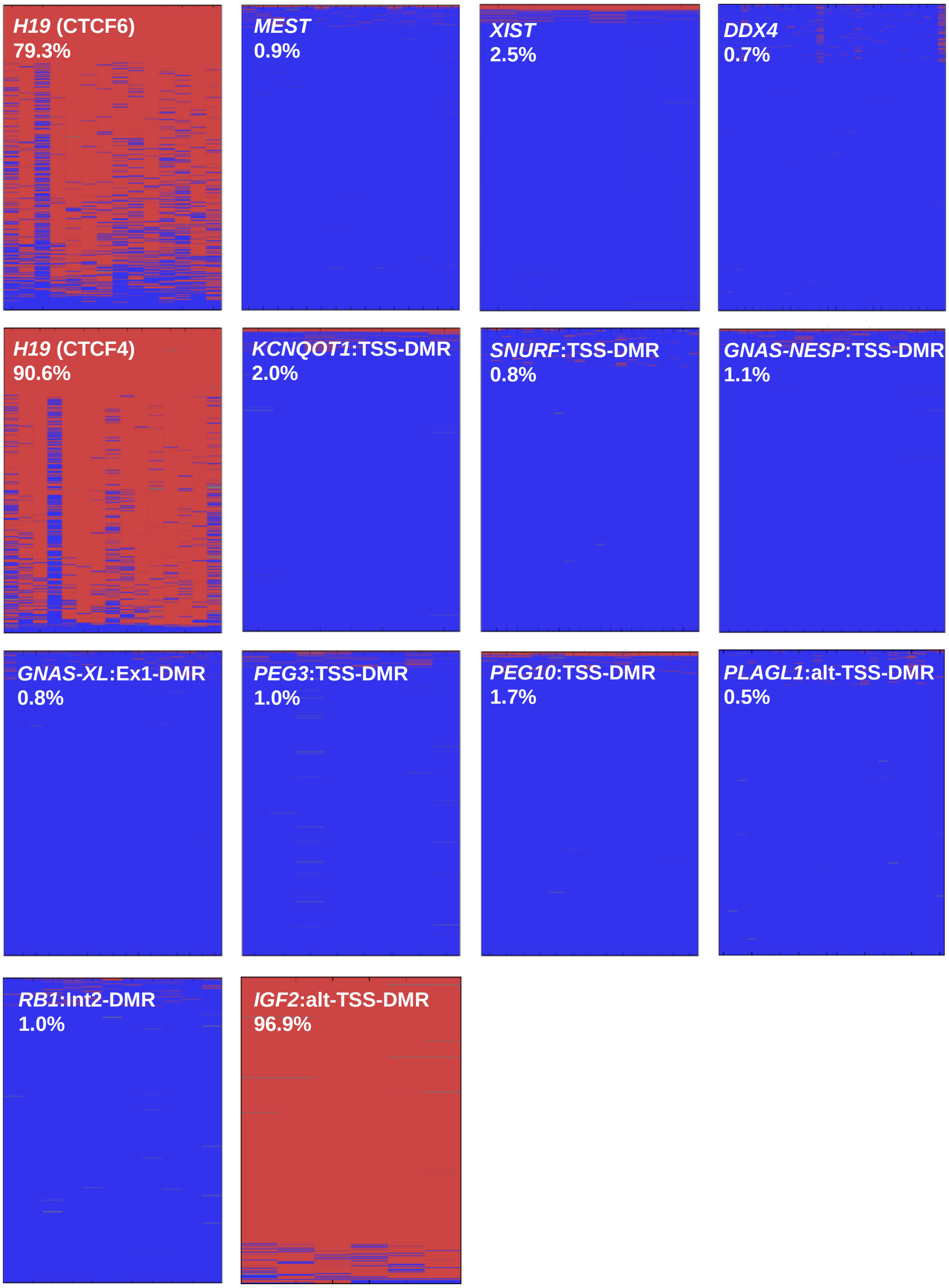

Fig. S8: Deep bisulfite sequencing results of the AMO sample SOAT6 with atypical *H19* methylation pattern.

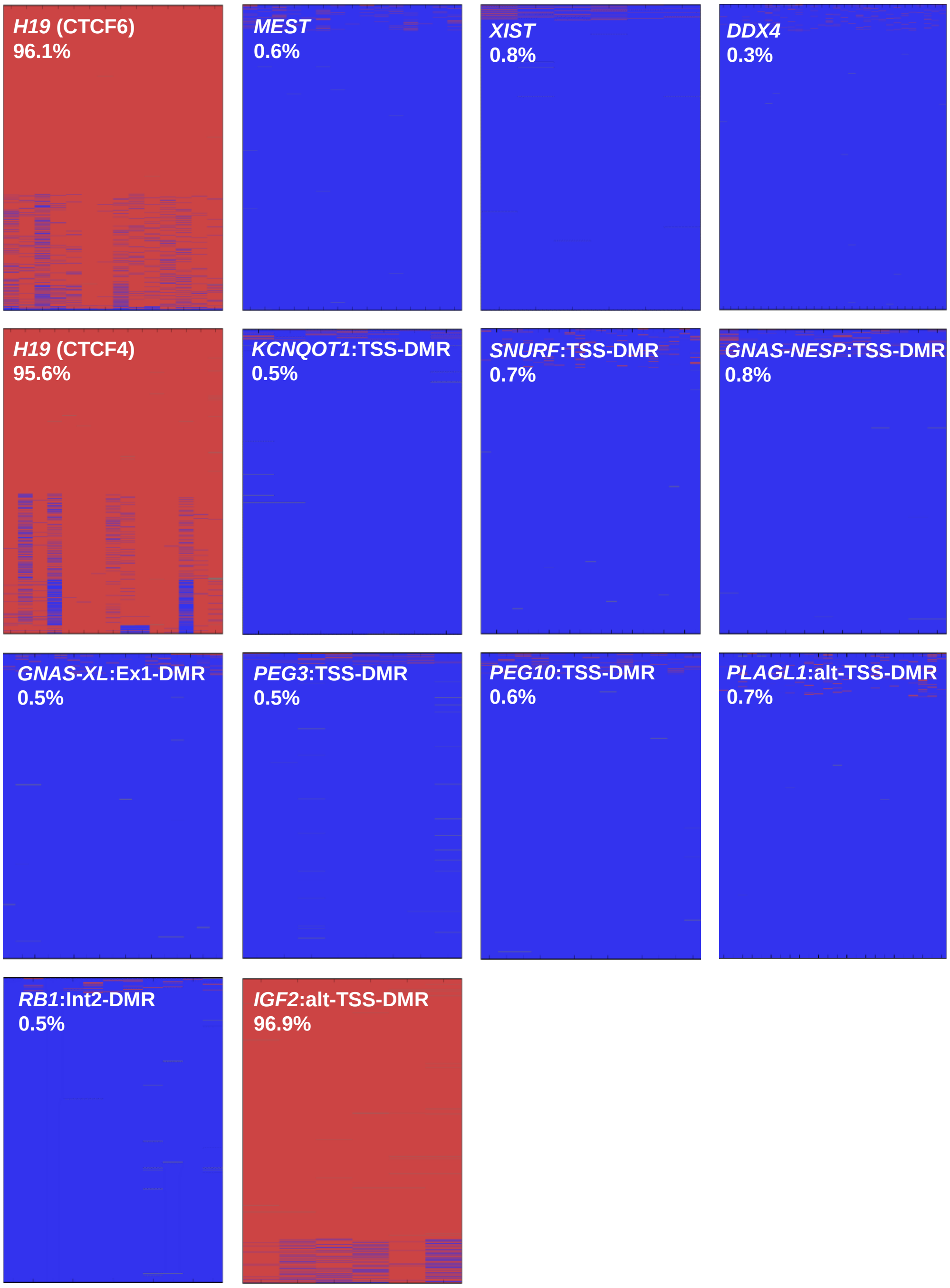

Fig. S9: Deep bisulfite sequencing results for a representative NC sample.

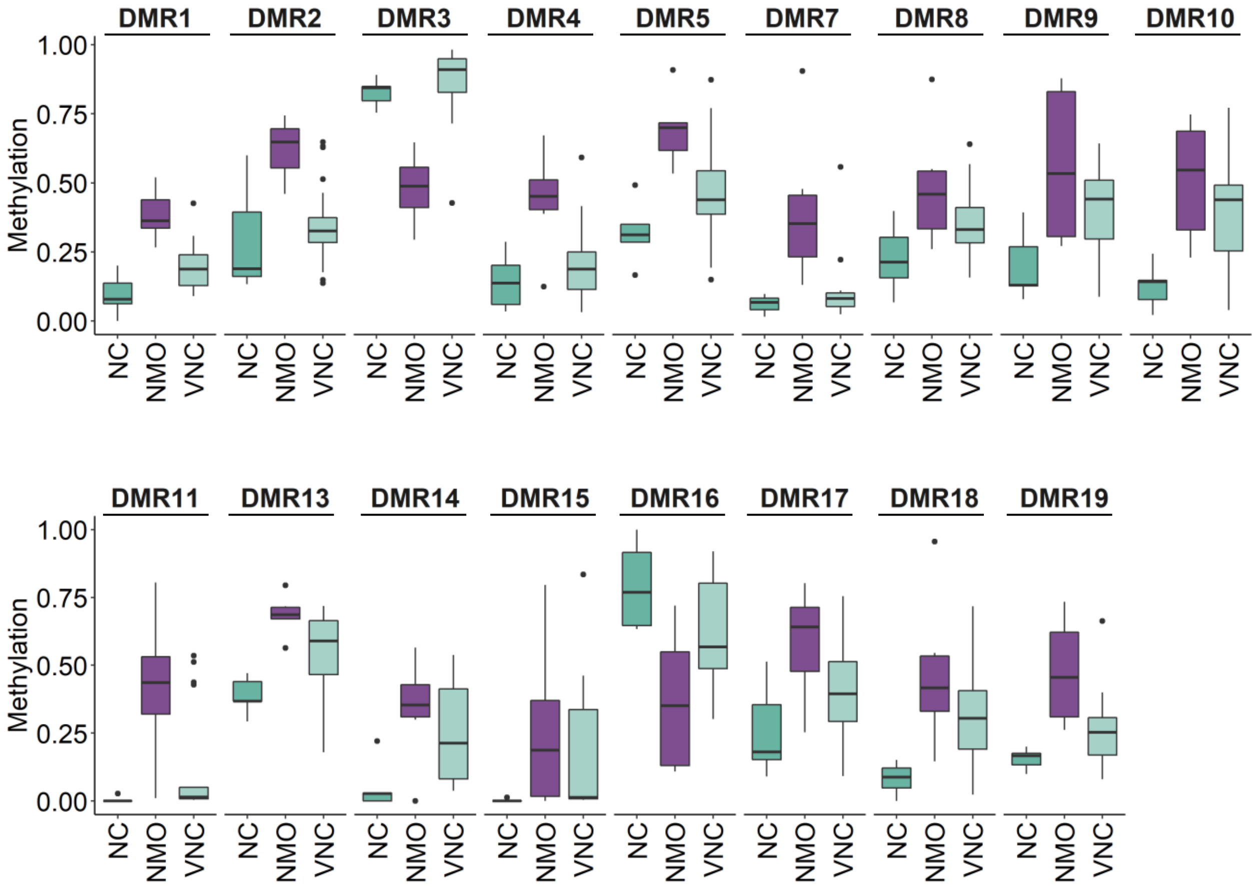

Fig. S10: Box plots showing for the 17 validated DMRs the distribution of the WGBS mean methylation values for the subset of CpGs covered by the targeted DBS approach (NC, teal, n = 5; NMO, purple, n = 6; Additional file 1: Table S9) and the targeted DBS methylation values for the validation NC samples (VNC, light teal, n = 20; Additional file 1: Table S11). Box plots elements are defined as follows: center line: median; box limits: upper and lower quartiles; whiskers: 1.5× interquartile range; points: outliers.

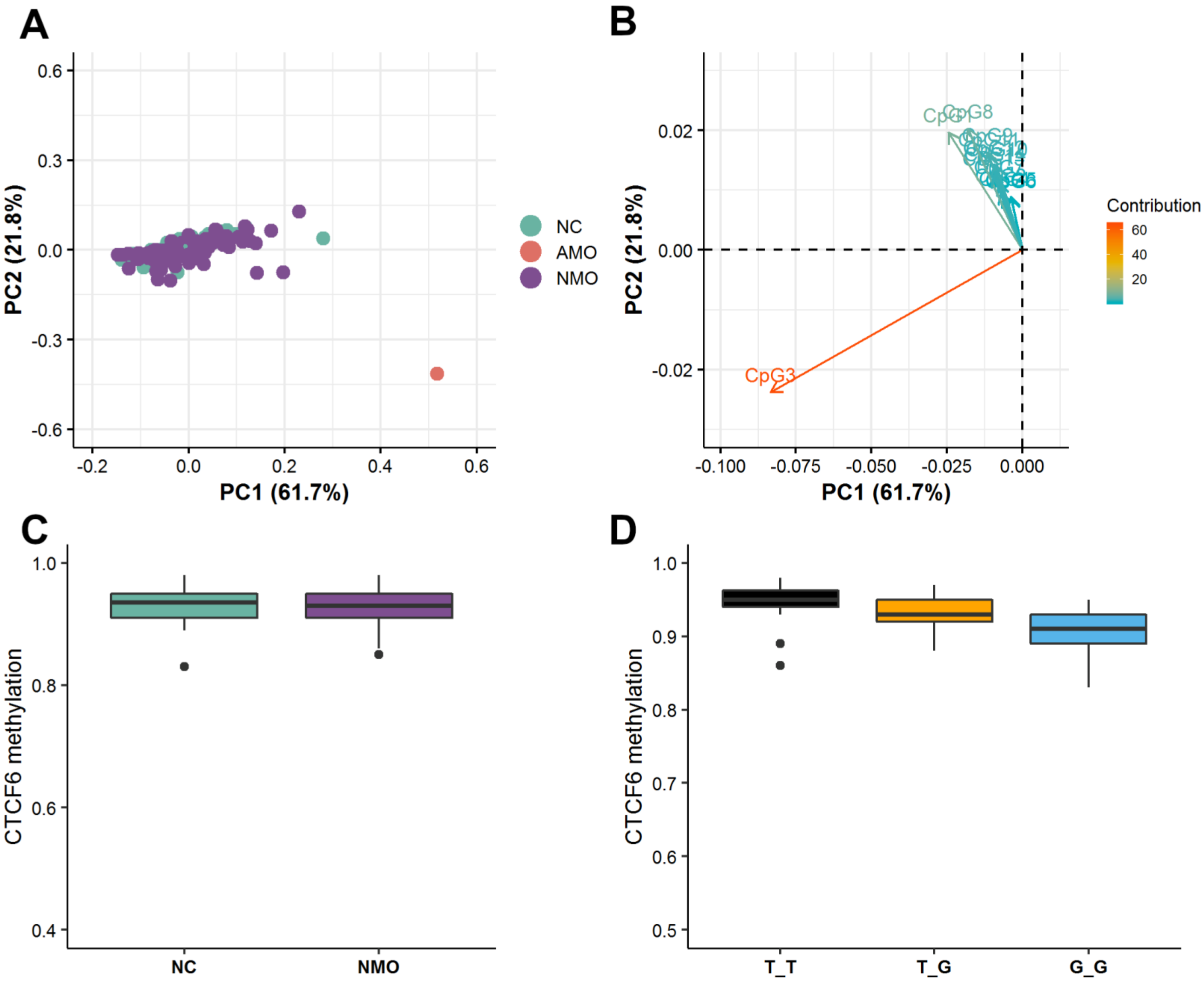

Fig. S11: A) Principal component analysis (PCA) of the 14 CpG sites in the *H19* locus obtained by DBS for the 40 normal controls (NC, teal), 77 normally methylated oligozoospermic (NMO, purple) and one abnormally methylated oligozoospermic (AMO) (Additional file 1: Table S13). B) Contribution of the variables (14 CpG sites) to the principal components. C) Comparison of the *H19* CTCF6 (CpG2-4) methylation between NC (n=40) and NMO (n=77) groups. Wilcoxon rank sum test, p-value = 0.9216. D) Comparison of the *H19* CTCF6 (CpG2-4) methlyation between individuals grouped according to the SNP rs2071094 genotype. Kruskal-Wallis, p-value = 1.328×10^{-10}. Pairwise comparisons using Wilcoxon rank sum test: GG vs TG p-value = 3.7×10^{-5}; GG vs TT p-value = 9.0×10^{-9}; TG vs TT p-value = 2.1×10^{-5} (Additional file 1: Table S15).
